# A fast detection of fusion genes from paired-end RNA-seq data

**DOI:** 10.1186/s12864-018-5156-1

**Published:** 2018-11-01

**Authors:** Trung Nghia Vu, Wenjiang Deng, Quang Thinh Trac, Stefano Calza, Woochang Hwang, Yudi Pawitan

**Affiliations:** 1 Department of Medical Epidemiology and Biostatistics, Karolinska Institutet, Nobels väg 12A, Stockholm, 17177 Sweden; 2 Department of Computational Sciences and Engineering, VNU University of Engineering and Technology, Xuan Thuy, 144, Hanoi, 84024 Vietnam; 3 Department of Molecular and Translational Medicine, University of Brescia, Viale Europa, 11, Brescia, 25125 Italy; 4 Data Science for Knowledge Creation Research Center, Seoul National University, Seoul, 151-747 South Korea

**Keywords:** Fusion gene, RNA sequencing, Quasi-mapping, Fusion equivalence class

## Abstract

**Background:**

Fusion genes are known to be drivers of many common cancers, so they are potential markers for diagnosis, prognosis or therapy response. The advent of paired-end RNA sequencing enhances our ability to discover fusion genes. While there are available methods, routine analyses of large number of samples are still limited due to high computational demands.

**Results:**

We develop FuSeq, a fast and accurate method to discover fusion genes based on quasi-mapping to quickly map the reads, extract initial candidates from split reads and fusion equivalence classes of mapped reads, and finally apply multiple filters and statistical tests to get the final candidates. We apply FuSeq to four validated datasets: breast cancer, melanoma and glioma datasets, and one spike-in dataset. The results reveal high sensitivity and specificity in all datasets, and compare well against other methods such as FusionMap, TRUP, TopHat-Fusion, SOAPfuse and JAFFA. In terms of computational time, FuSeq is two-fold faster than FusionMap and orders of magnitude faster than the other methods.

**Conclusions:**

With this advantage of less computational demands, FuSeq makes it practical to investigate fusion genes in large numbers of samples. FuSeq is implemented in C++ and R, and available at https://github.com/nghiavtr/FuSeqfor non-commercial uses.

**Electronic supplementary material:**

The online version of this article (10.1186/s12864-018-5156-1) contains supplementary material, which is available to authorized users.

## Background

Gene fusion, one type of structural chromosome rearrangements, has been found to play important roles in carcinogenesis [1, 2]. It is closely associated with an increase of chimeric proteins, with cancer risk and with tumor phenotypes, all of which have potentials for clinical translation [2]. Fusion genes are reported in different types of cancers such as breast cancer [3, 4], lung cancer [5], melanoma [6] and glioma [7]. A fusion gene ETV6-RUNX1 was recently discovered in approximately 20%-25% of childhood acute lymphoblastic leukemia [8]. A further discussion of gene fusion in cancer can be found in a recent review [2].

The advent of RNA sequencing (RNA-seq) technology allows us to efficiently discover novel fusion genes. Many tools have been developed for detecting fusion transcripts using RNA-seq data, and their comparisons are available in several recent publications [9, 10]. These methods use various approaches, but generally include three main steps: (i) read alignment, (ii) fusion candidate detection and (iii) false positive elimination. Read alignment is usually done by standard read alignment methods in RNA-seq, such as TopHat-Fusion [11], SnowShoe-FTD [12], EricScript [13], JAFFA [14], or by its own method as FusionMap [15]. To determine fusion candidates, most of the methods use discordant reads such as spanning read pairs and/or split reads. Spanning reads contain one read located in different genes, while split reads indicates a single read overlapping on two different genes. The final step contains filtering and/or scoring systems to remove false positive fusion candidates. This step varies from method to method and has been summarized in several reviews [9, 10].

Most of the current fusion detection methods require significant computational demands. As reported recently [9] for 15 fusion detection methods, including BreakFusion [16], Chimerascan [17], defuse [18], EricScript [13], FusionCatcher [3, 19], FusionHunter [20], FusionMap [15], FusionQ [21], JAFFA [14], MapSplice [22], PRADA [23], ShortFuse [24], SnowShoes-FTD [12], SOAPfuse [25], TopHat-Fusion [11], they require from 7 to 240 h to analyse a single prostate cancer sample containing ∼118M 100bp-long read pairs. This makes it impractical to perform routine fusion gene detection in datasets with a large number of samples. To address this, we develop FuSeq, a novel fusion detection method utilizing a recent quasi-mapping method for alignment that is substantially faster than traditional alignment methods [26]. FuSeq consists of two separate pipelines based on mapped read-pairs (MR) and junction split-reads (SR) that are combined in the final step. For the MR pipeline, FuSeq introduces a new concept of fusion-equivalence class to generate fusion candidates. In the SR pipeline, fusion candidates are collected from split reads where two different genes share the same read, and each gene has at least k-mer mapped bases. In addition, various filters and statistical tests are applied to the fusion candidates, primarily for false-positive reduction. We apply FuSeq to four validated datasets and show that it outperforms commonly used methods Tophat-fusion, SOAPfuse and JAFFA in sensitivity, specificity and discovery operating characteristics, while FuSeq is orders of magnitude faster in computational time.

## Methods

**Fig. 1:**
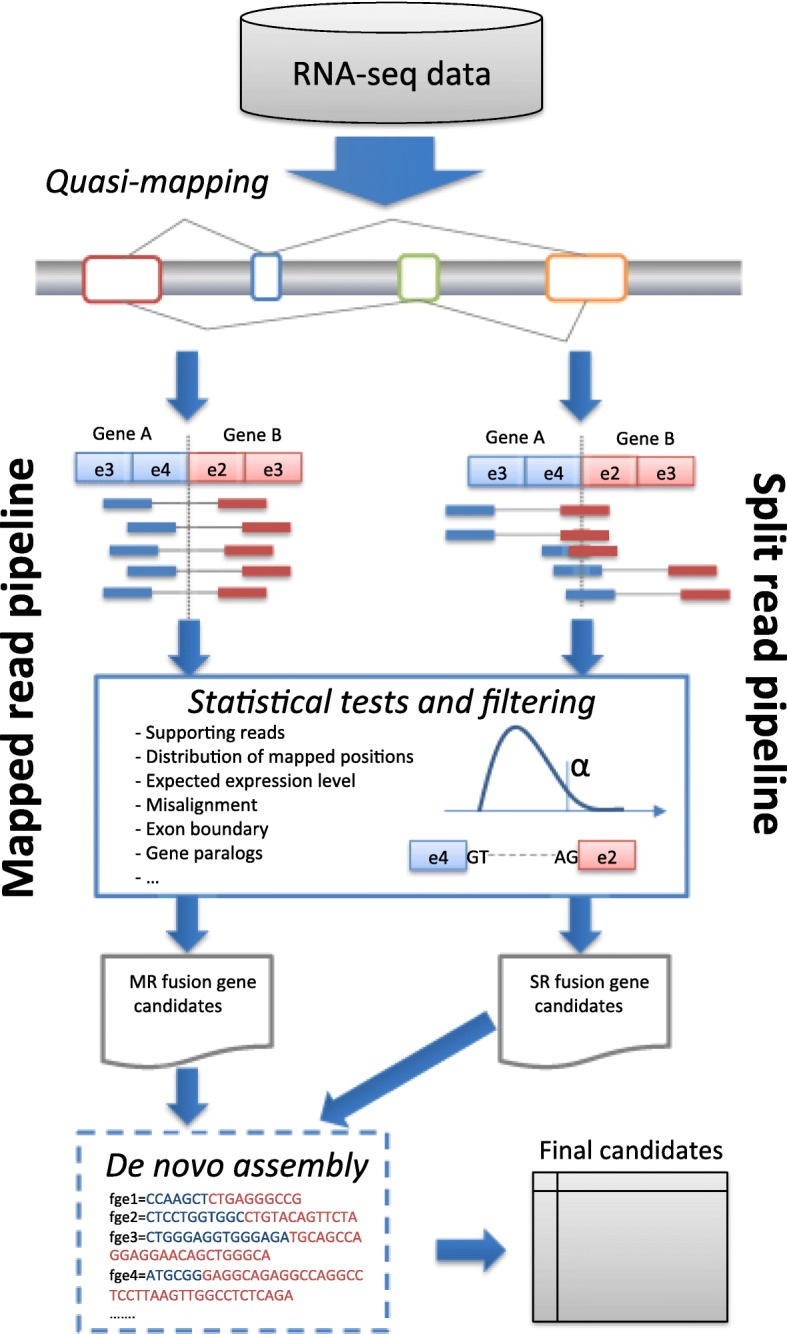
FuSeq pipeline for fusion gene detection: quasi-mapping of read pairs to extract mapped reads and split reads; statistical tests and filtering to eliminate false positive fusion genes; collecting and merging fusion gene candidates from mapped reads and split reads; de novo assembly to verify and determine fusion sequences; and exporting information of final candidates to files

The pipeline of the proposed fusion detection is presented in Fig. 1. The key steps are: (i) quasi-mapping to detect mapped reads and split reads of fusion candidates, and (ii) statistical tests and filtering, separately for mapped-read pipeline and split-read pipeline to eliminate likely false positives. Before exporting the final results, de novo assembly can be used as an extra step to verify and determine the fusion sequence. The details of each phase are presented in the following sections.

### Mapped reads and split reads

We utilise the quasi-mapping from Rapmap to generate mapped reads and split reads for FuSeq. The split reads and mapped reads in FuSeq are determined depending on k-mer length *k* and read-length *r* used in the quasi-mapping. A mapped read is from a read-pair where each read is completely or mostly (with length >=*r*−*k*−1) mapped to a different gene. In split reads, a single read is partially mapped in both genes and the mapped sequences must have a length >=*k*. Additional file 1: Figure S1 intuitively demonstrates different cases of mapped reads and split reads. It is worth noting that, by definition, mapped reads in FuSeq will span over the fusion junction-break.

### Fusion-gene candidates from split reads

In the quasi-mapping of Rapmap, each read is mapped to a transcriptome stored in a k-mer index system. The result of a quasi-mapping for a read is a list of k-mers (ordered from left to right) of the read. For each split read, we extract the important mapping information of the k-mers at the first and the last of the list for downstream analysis. The information includes mapping directions, query positions of the k-mers, mapped transcripts and corresponding genes, mapped positions of the transcripts, and the mapped position of the other read of the pair. The pair of the split read must be mapped to the same transcript on either side of the fusion. Additional file 1: Figure S2 presents the details of the data structure extracted from split reads. All possible split reads are collected to input to statistical tests and filtering steps.

### Fusion-gene candidates from mapped read

Since the number of mapped reads are significantly higher than the number of split reads, to speed up calculation, we introduce a novel concept “fusion equivalence class” to organize and generate fusion-gene candidates.

#### Fusion equivalence class

We first explain the concept of fusion equivalence class, which is motivated by the transcript equivalence class used for transcript quantification by Patro et al. [27]. For a given read pair (*r*_1_,*r*_2_), using quasi-mapping from Rapmap [26], we extract *T*_1_ and *T*_2_ as the sets of transcripts that *r*_1_ and *r*_2_ map to, respectively. We define *S*≡*T*_1_∩*T*_2_ as the concordant transcript set between *T*_1_ and *T*_2_. We denote *S*_1_≡*T*_1_−*S* and *S*_2_≡*T*_2_−*S* as the sets of discordant mapped transcripts of *r*_1_ and *r*_2_, respectively. From now on, we define a fusion transcript (ftx) as an ordered combination of two transcripts tx_*u*_ and tx_*v*_ belonging to the discordant mapped transcript sets:

1 $$ \text{ftx}(u,v)=(\text{tx}_{u}, \text{tx}_{v}),   $$

where tx_*u*_∈*S*_1_ and tx_*v*_∈*S*_2_.

Additional file 1: Figure S3 displays a simple example where *S*_1_={tx_1_,tx_2_} and *S*_2_={tx_3_,tx_4_,tx_5_}. From *S*_1_ and *S*_2_, there are six possible fusion candidates generated from combination between the transcripts in *S*_1_ and the transcripts in *S*_2_: ftx_1_=(tx_1_,tx_3_),ftx_2_=(tx_1_,tx_4_),ftx_3_=(tx_1_,tx_5_),ftx_4_=(tx_2_,tx_3_),ftx_5_=(tx_2_,tx_4_),ftx_6_=(tx_2_,tx_5_).

For simplicity here we denote each fusion transcripts with a single index, but there is no conflict with the notation in formula 1. We are always able to refer a fusion transcript to an ordered combination of two transcripts, for example ftx_1_=ftx(1,3)=(tx_1_,tx_3_). Thus, each read-pair maps to or generates a set of fusion candidates (which could be empty). Two read-pairs are said to be equivalent if (and only if) they map to the same set of fusion transcripts; this means we can group/partition the read-pairs into equivalence classes using the corresponding set of fusion transcripts as the group index. Thus we set up the fusion equivalence (feq) classes – each indexed by the set of potential fusion transcripts (ftx_1_&*hellip*;ftx_*n*_) – such that every read-pair in a feq must map exactly to all the fusion transcript candidates that defines the feq.

In the example above, a read pair (*r*_1_,*r*_2_) belongs to a fusion equivalence class feq consisting of six fusion transcripts and contributes one count to that equivalence class.

Naturally, from a single fusion transcript ftx(*u*,*v*) we can also derive a fusion gene (fge) as a combination of two genes: gene_*A*_ and gene_*B*_ as follows:

2 $$\begin{array}{@{}rcl@{}} \text{fge}=\left(\text{gene}_{A},\text{gene}_{B}\right)\sim\left\lbrace\left(\text{tx}_{u},\text{tx}_{v}\right)\right\rbrace,  \end{array} $$

where tx_*u*_ is a transcript of gene_*A*_, and tx_*v*_ is a transcript of gene_*B*_. Thus, a fusion gene is connected to fusion equivalence classes through its corresponding fusion transcripts. If the transcript pair (tx_*u*_,tx_*v*_) belongs to feq_*j*_, then we say feq_*j*_ supports the fge.

We denote the set of fusion equivalence classes as FEQ={feq_1_..feq_*M*_} with a set of corresponding numbers of supporting read pairs *C*={*c*_1_..*c*_*M*_}, the set of fusion transcript as FTX={ftx_1_..ftx_*N*_} and the set of fusion gene FGE={fge_1_..fge_*K*_}. The FEQ table in Additional file 1: Figure S3 also shows the relationship between FEQ, FTX and FGE. The first and the second rows of the table presents fusion gene and its fusion transcripts. Each following row presents a single fusion equivalence class. The binary value indicates the absence/presence of ftx_*i*_ in feq_*j*_. The last column shows the number of fragments supporting the corresponding fusion equivalence class. From the table, we extract the number of fragments supporting a fusion gene by summing up the *c*_*j*_ of its supporting fusion equivalence classes, that is those feq including any ftx included in the fge_*k*_ set as follows

3 $$\begin{array}{@{}rcl@{}} \text{supportCount}(\text{fge}_{k})\equiv \sum\limits_{j} c_{j},  \end{array} $$

where summation over all *j* such that feq_*j*_’s support fge_*k*_. We also can compute the number of fragments supporting a fusion transcript supportCount(ftx_*i*_) by the same formula with the *c*_*j*_ supporting fusion transcript ftx_*i*_.

If many fusion genes share the same fusion equivalence classes then this indicates that the supporting reads from these fusion equivalence classes are not uniquely mapped to a single fusion genes. To compensate this issue, we correct the supporting counts of the fusion genes by adding weights of fusion genes in the fusion equivalence class. For simplicity, we set the weights of all fusion genes of a fusion equivalence class to be equal. Thus, the corrected count is computed as follows

4 $$\begin{array}{@{}rcl@{}} \text{correctedCount}(\text{fge}_{k})\equiv \sum\limits_{j} c_{j}*w_{k,j}  \end{array} $$

where *w*_*k*,*j*_=1/|feq_*j*_| and |feq_*j*_| is the number of fusion transcripts.

We also discard all fragments with a fusion equivalence class containing two transcripts from the same gene. Finally, a list of fusion-gene candidates is extracted from the table of fusion equivalence classes for further analysis.

From hereon the term ‘fusion transcript’ is defined in relation to the fusion equivalence classes, which is not necessarily a transcript generated from a fusion event. The ‘fusion gene’ indicates a fusion event occurring between two separate genes, consequently generating a fusion transcript. The aim of our method is to detect a fusion event between two genes, so ‘fusion gene’ is reported as the final result.

### Statistical tests and filtering

Several statistical tests and filtering criteria are applied to limit false-positive fusion-gene candidates. We divide the filters into three main categories: (i) general features of fusion genes; (ii)sequence similarity of constituent genes; (iii) positional distribution of the supporting reads. The applications of the filters and tests might be different from mapped reads to split reads. The details of the filters for practical implementation in each pipeline are supplied in the Supplementary report.

#### General features of fusion genes

We limit fusion genes to common situations, for example: in selected chromosomes 1-22, X and Y, constituent genes coming from protein-coding genes, large enough distance between constituent genes, and sufficient supporting read count. We also do not allow ’inverted fusion’ that if a fusion gene fge(gene_*A*_,gene_*B*_) is expressed, the inverted direction fusion gene fge(gene_*B*_,gene_*A*_) is not likely expressed. The inverted fusion gene created by exchanging the roles between 5-prime gene and 3-prime gene from one fusion gene. This can create circular fusions that are likely false positives.

#### Sequence similarity of constituent genes

Different genes with highly similar sequences are often listed in fusion-gene candidates but they are likely false positive. The similarity is frequently observed between a gene and its paralogs or its known read-through (conjoined) genes, that can be collected from the reference database. Since mapped reads do not generally contain junction-break information, we use strict criteria for the mapped read pipeline to reduce false positives. In particular, we do not expect many supporting read pairs to be shared between fusion genes. Furthermore, we utilize the equivalence classes to discover all the possible sequence similarities between two genes that will be used as an extra paralog reference.

Specifically, we first use RNASeqReadSimulator tool (http://alumni.cs.ucr.edu/~liw/rnaseqreadsimulator.htm) to generate a paired-end simulation sample, where the expected read counts of all transcripts of the transcriptome are equal and high enough, herein 1000 read counts. Then, transcript equivalence classes of the simulation sample are generated using Rapmap [26]. Since all transcripts are expressed, two transcripts with similar sequence regions have to appear in at least one equivalence class. Finally, the transcripts are mapped to their corresponding genes to determine which genes share similar sequences.

#### Positional distribution of supporting reads

For each read pair (*r*_1_,*r*_2_) of mapped reads, we collect all start positions of *r*_1_ and *r*_2_ mapped to the annotation reference. Thus, for each fusion gene (gene_*A*_,gene_*B*_), we are able to compute the distributions of the start positions (startPos) of the supporting reads to gene_*A*_ and gene_*B*_. Similarly we get the distributions of start positions of the k-mer sequences mapped to the genes from the split reads. For simplicity, we call them positional distribution. If the start positions of two single reads are very close to each other, they are likely duplicated (quasi-duplicated). This feature is similar to the criteria of patterns of short reads mentioned in [3, 13]. Collections of positional distribution can be used to estimate the fragment length generating a read pair. Then statistical tests for fragment length are used to remove outliers and eliminate false positives. In addition, breaking points and exons information of each site can be estimated from the positional distribution. Thus, FuSeq can allow to check the satisfactions of popular splicing sites such as GT-AG, GC-AG and AT-AC. Moreover, if the distance between two breaking points from the same chromosome (junctionDistance) is too close to each other, the fusion-gene candidate is likely a false positive.

#### Other statistical tests and filtering

In general, paralogs can easily produce false-positive candidates, but these candidates will have too many sequence similarities. Thus we check the length of the overlapping mapped sequence between two genes in a split read based on the first and the last k-mers. Moreover, a split read is deemed a misalignment if >85*%* of the read sequence is fully mapped to either 3’ transcript or 5’ transcript. Finally, the consistency between expression level of mapped reads and split reads supporting a fusion gene is tested. We also report (but not eliminate) the genes relating to mitochondrial translation, cytosolic ribosomal subunit and ribonucleoprotein, which are filtered out in some fusion detection methods [3, 19].

### De novo assembly

If the number of supporting read pairs of a fusion gene is large enough, then it is useful to perform de novo assembly to build a sequence-contig capturing the fusion. This step is optional in FuSeq and any denovo assembly tools such as Trinity [28], Oases [29], Trans-ABySS [30], SOAPdenovo-Trans [31] can be used. To get a computationally efficient procedure, the de novo assembly is done as follows. First, we extract all read pairs supporting all fusion-gene candidates from the previous stage of the pipeline. *This produces a small set of reads, so the assembly computation is trivial.* These read pairs (both mapped reads and split reads) are used as input to a de novo assembly tool to construct the contigs; in practice we use Trinity [28]. Next, we consider these contigs as the reference for Rapmap [26] and do quasi-mapping for the read pairs to the reference. From the mapping results we can associate the contigs to the candidate fusion genes.

### Final fusion-gene candidates

The final set of fusion-gene candidates is combined from the candidate lists of mapped-read and split-read pipelines. It is worth noting that the fusion gene candidates from mapped read might have no supporting split reads, and vice versa. The score of each fusion gene is the sum of the corrected count of the supporting mapped-reads and split-reads. In FuSeq, scores of final fusion gene candidates must be at least 3. In the final set, fusion genes are ranked according to their scores.

### Implementation

In our implementation, we use the genome reference and annotation from Ensemble version GRCh37.75. The method was implemented in C/C++ for extraction of split reads and fusion equivalence classes of mapped reads, combined with R language for downstream analysis. FuSeq software is available for non-commercial use at https://github.com/nghiavtr/FuSeq. User guides with practical examples are also provided in the website.

### Materials

We illustrate the applications of FuSeq to four publicly available and validated real datasets.

#### Breast-cancer dataset

There are 6 samples from 4 breast-cancer cell lines (BT-474, SK-BR-3, KPL-4 and MCF-7) [3], where BT-474 and SKBR3 have two samples. The samples contain 14-42M paired-end reads of 50bp long, using Qiagen PCR purification kit in library preparation following sequenced by 1G Illumina Genome Analyzer 2X. There are 27 validated fusion genes from the original study [3], and extended to a total of 99 in later publications [3, 4, 14]. For clarity we will separately call these two validated versions as TP27 and TP99 datasets. They highlight the difficulty in assessing a method when there is no real gold standard. For example, some fusion candidates that are not validated in TP27 dataset turn out to be true positives in the TP99 dataset.

#### Melanoma dataset

This dataset has 6 samples (501-MEL, M000216, M000921, M010403, M980409 and M990802) from melanoma patients [6] including 8-16M paired-end reads of 50bp long. The library was prepared using the SuperScript Double-Stranded cDNA Synthesis kit (Invitrogen) before sequenced by Illumina Genome Analyzer II. A total of 11 fusion genes has been validated in this dataset.

#### Glioma dataset

This dataset has 13 patient samples from a glioma study [7] (SRA accession SRP027383): SRR934744, SRR934746, SRR934774, SRR934868, SRR934871, SRR934875, SRR934887, SRR934902, SRR934915, SRR934918, SRR934929, SRR934930, SRR934947. The glioma dataset contains 15-35M paired-end reads of 101bp. The library was prepared using using SuperScript III reverse transcriptase (Invitrogen) and sequenced by the Illumina HiSeq 2000 platform. A total of 31 fusion genes has been validated in this dataset.

#### Spike-in dataset

This dataset contains 9 synthetic spike-in fusion genes titrated into the cell line COLO-829 [32] with ten different abundances, each abundance has one duplicate. The final 20 RNA-seq samples have 72-180M paired-end reads of 100bp long. The library is prepared by the TruSeq Stranded mRNA LT Sample Prep Kit, later sequenced using Illumina HiSeq2500. Thus, the strandness of the read is kept in this dataset. In this dataset, sample SRR1659964 with the medium concentration (–6.17 log10(pMoles)) was compared to other dataset recently (Liu et al., 2015), so it is also considered in a separate comparison.

### Competing tools and evaluation

We select TopHat-Fusion, JAFFA and SOAPfuse as the main tools for comparisons. TopHat-Fusion is a widely used fusion gene detection tool, JAFFA is one of the most recent and top performing methods, and SOAPfuse is the best method in the recent comparison [9]. We compare the performance in terms of sensitivity, specificity and computational time. We utilize results from a recent study [14], because it contains performances of JAFFA, SOAPfuse and TopHat-Fusion for the breast-cancer dataset and the glioma dataset. The original study of the spike-in dataset [32] is used for the comparison of all 20 samples with TopHat-Fusion. We also use a comparative study of fusion detection methods [9], since it contains information of the melanoma dataset and sample SRR1659964 of the spike-in dataset. Finally, for comparison of computational time, we select FusionMap [15], the fastest method from the comparative study, and a recent method TRUP [5], which demonstrated similar computational performances to FusionMap and TopHat-fusion. We use the version of FusionMap migrated in the Oshell pipeline at http://www.arrayserver.com/wiki/index.php?title=Oshell since all older versions of FusionMap are inactive. We use version TRUP _2015-21-05 from the TRUP webpage https://github.com/ruping/TRUP/.

Since the performances of methods depend on the parameter settings [9], for fair comparisons, we set the parameters in our method similar to the corresponding values from the previous comparisons. For example, similar to the other methods, FuSeq also depends on the minimum number of supporting spanning reads (mapped reads in FuSeq) to remove false positives. Thus, we set this value as 2 for the comparison of the breast-cancer dataset, the glioma dataset and all 20 samples of spike-in dataset, and 3 for the comparison of the melanoma dataset and sample SRR1659964 of the spike-in dataset. The minimum supporting reads is set to 1 as common. We also implement de novo assembly to verify the true positives in a few samples from the datasets. Since there are no particular settings of FusionMap and TRUP for the minimum number of supporting spanning reads or spliting reads, these criteria are not applicable. We also used GSNAP (default) for the mapper of TRUP since it gets more sensitivity than STAR [5]. For other setting parameters of FusionMap and TRUP, the default values are used. Since the breast-cancer dataset and the melanoma dataset have short reads (50 bp), a low value (=1) is set for the parameter *consisCount* (number of consistent read pairs with discordant mapping) of the step “runlevel 3” as the suggestion of the tool. However, using either the default value (*consisCount*=5) or the suggested value (*consisCount*=1), TRUP reported no fusion gene candidates. Since we are not sure if this is the proper result, and we could not find any obvious solutions, we do not report the performance metrics (“-”) for TRUP for these two datasets (Table 1).

**Table 1:** Fusion discoveries in the cancer datasets. The results for TopHat-Fusion, SOAPfuse and JAFFA are collected from a recent study [14]

		FusionMap	TRUP	TopHat-Fusion	JAFFA	SOAPfuse	FuSeq
Breast cancer (TP27)	Total	47	0	261	42	61	53
	TP	12	0	24	20	24	22
	Recall	0.44	-	0.89	0.74	0.89	0.81
	Precision	0.26	-	0.09	0.48	0.39	0.42
	F1	0.32	-	0.17	0.58	0.55	0.55
	*P*-value	0.32	-	9.1e-06	0.71	1	-
Breast cancer (TP99)	Total	47	0	261	42	61	53
	TP	22	0	35	28	41	36
	Recall	0.22	-	0.35	0.28	0.41	0.36
	Precision	0.47	-	0.13	0.67	0.67	0.68
	F1	0.30	-	0.19	0.40	0.51	0.47
	*P*-value	0.32	-	1.1e-08	1	1	-
Melanoma	Total	19	0	29	4	108	21
	TP	3	0	4	2	10	7
	Recall	0.27	-	0.36	0.18	0.91	0.64
	Precision	0.16	-	0.14	0.5	0.09	0.33
	F1	0.20	-	0.2	0.27	0.17	0.44
	*P*-value	0.48	-	0.32	0.62	0.02	-
Glioma	Total	191	209	308	904	299	188
	TP	28	20	29	30	22	29
	Recall	0.90	0.65	0.94	0.97	0.71	0.94
	Precision	0.15	0.10	0.09	0.03	0.07	0.15
	F1	0.25	0.17	0.12	0.05	0.13	0.26
	*P*-value	0.89	0.13	0.09	5.5e-08	0.02	-

We select the best result from the different runs ofcomparison. TP= true positive fusion genes, Total= total discovered fusion-gene candidates, *P*-value = two-sided *p*-value of Fisher’s exact test of the difference in precision between FuSeq vs each of the other methods

In addition, to be able to generate split reads in FuSeq, the k-mer length must be less than a half of read length. The default setting of k-mer length 31 in Rapmap is not suitable for dataset with short reads (50 bp read long). Therefore, we set this value as 21 for the short read datasets (breast-cancer and melanoma datasets with 50bp read long) and as 31 for long read datasets (gliomas and spike-in datasets with 100 bp read long).

## Results

### Illustration

**Fig. 2:**
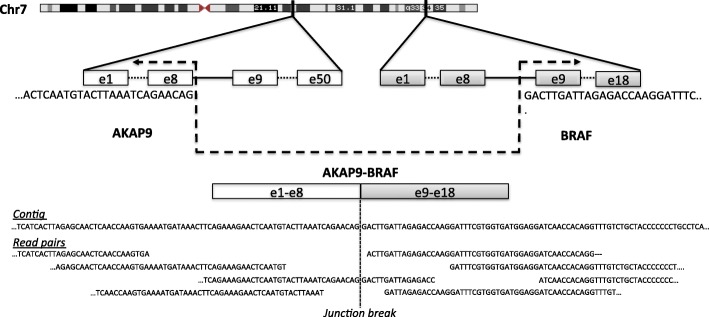
Discovery of AKAP9-BRAF fusion by FuSeq in the spike-in dataset SRR1659964. Also shown are the contig from the de novo assembly and a sample of 4 read-pairs near the junction break

Figure 2 shows one example of a true fusion event involving AKAP9-BRAF genes in chromosome 7, discovered by FuSeq in the spike-in dataset SRR1659964. The full data contains 93.9M read-pairs, and the fusion gene contains 3318bp and 1158bp from AKAP9 and BRAF, respectively. In Fuseq output, the fusion is supported by 60 read-pairs. It is a highly confident fusion, as it is also supported by a contig constructed from the de novo assembly; this contig is 639bp long with 252bp belonging AKAP9.

### Discovery performance

The discovery operating characteristics for the breast-cancer, melanoma and glioma datasets are presented in Table 1. The results reported here are pooled from all the samples of each dataset. We use recall, precision and F1 score for the comparison. Recall or sensitivity is defined as the ratio between the discovered true positives and the total validated fusion genes. Precision is the ratio between the discovered true positives and the total fusion candidates discovered by the fusion-detection methods. The precision conveys the specificity of the methods. F1 score or F-measurement is a balanced metric between precision and recall, and is calculated by

$$\text{F1}=\frac{2*\text{precision}*\text{recall}} {\text{precision + recall}}. $$

The list of validated true positives in each dataset is likely incomplete; assuming this list is a random subset of all true positives, the reported recall is unbiased, but precision is only a lower bound of the true values.

For the breast-cancer data, FuSeq discovers 22 of 27 validated fusions in TP27, and 36 of 99 extended validated fusions in TP99. Compared to FusionMap and TopHat-Fusion, FuSeq discovers more true positives and has a smaller candidate list. JAFFA has the smallest final fusion-gene list, but it loses many validated fusion genes. The F1 score of FuSeq is equal to SOAPfuse and slightly smaller than that of JAFFA in TP27 (0.55 vs 0.58). However, F1 scores of both FuSeq and SOAPfuse are significantly greater in TP99 (0.47 and 0.51 vs 0.40). As mentioned in the “Methods” section, TRUP reported no fusion gene candidates, thus we consider its metrics as not available (“-”) for the comparisons in this dataset, and similarly in the melanoma dataset.

As described in a recent study [9], the melanoma dataset represents a difficult dataset for fusion-gene detection. Among 15 competing fusion gene detection tools, most of them find less than 6 validated fusion genes. FusionMap, TopHat-Fusion and JAFFA report 3, 4 and 2 true positives, respectively; this is shown in Table 1. SOAPfuse discovers most true positives (10) but introduces a lot of false positive fusion genes (recall=0.09). FuSeq reports 7 true positives and recommends 14 other candidates. FuSeq also has the best F1 score (0.44) as compared with the other methods.

In the glioma dataset, except for TRUP and SOAPfuse (with 20 and 22 discovered true positives, respectively), all the other methods discover most of validated fusion genes, 28 for FusionMap, 29 for both TopHat-Fusion and FuSeq, and 30 for JAFFA. However, FuSeq discovers the smallest fusion candidate set (188 candidates in total), which makes the F1 score of FuSeq higher than that of all other methods.

**Fig. 3:**
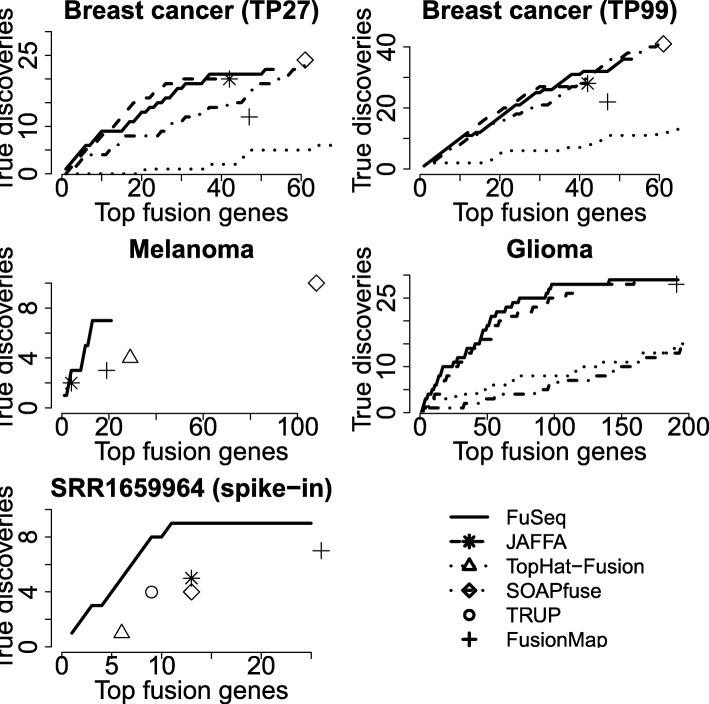
Comparisons of the operating characteristics in validated datasets. In each panel FuSeq result is given as a solid curve, and other results as dash curves or points. The results of JAFFA, SOAPfuse and TopHat-Fusion for breast-cancer and glioma datasets and melanoma and spike-in datasets are taken from Davison et al. [14] and Liu et al. [9], respectively. For purpose of visual comparison, the x-axis of the plots is limited mostly by the curves of FuSeq

For the spike-in dataset, due to the huge library sizes (72-180M read pairs), there are not many available results from current fusion detection methods for all 20 samples. The original publication of the spike-in dataset [32] reports the total number of spike-in fusion genes detected from TopHat-Fusion and ChimeraScan and SnowShoes-FTD tools for all 20 samples, but without information of other fusion-gene candidates. In this study, only 142, 80, 133 and 138 out of 180 true positives are discovered from FusionMap, TRUP, TopHat-Fusion and JAFFA, respectively (Table 2). As shown in the table, FuSeq obtains a much better result by missing only one true positive. We also find out another report [9] of the spike-in dataset, but only for a single sample SRR1659964 (-6.17 log10(pMoles) concentration). In the report, all 15 fusion gene detection tools are not able to detect all 9 spike-in fusion genes. From that report, JAFFA, SOAPfuse and TopHat-Fusion detect 5, 4 and 1 true positives respectively (Table 2). From the table, FusionMap and TRUP also discover 7 and 4 true positives, respectively. In contrast, FuSeq can detect all 9 spike-in fusion genes, and they are among the top 11 in fusion ranks (Fig. 3). FuSeq also shows the best F1 score (0.53) in this dataset.

**Table 2:** Fusion discoveries in the spike-in dataset. The results for TopHat-Fusion, SOAPfuse and JAFFA for sample SRR1659964 are collected from a recent study [9]

	One sample(SRR1659964)						20 samples		
	TP	Total	Precision	Recall	F1	*P*-value	TP	Other	*P*-value
FusionMap	7	26	0.27	0.78	0.40	0.78	142/180	283	0.04
TRUP	4	9	0.44	0.44	0.44	1	80/180	63	0.15
JAFFA	5	13	0.39	0.56	0.46	1	138/180	114	0.12
SOAPfuse	4	13	0.31	0.44	0.36	1	NA	NA	NA
TopHat-Fusion	1	6	0.17	0.11	0.13	0.66	133/180	925	1.6e-22
FuSeq	9	25	0.36	1	0.53	-	179/180	228	-

TP= true positive fusion genes, Other= unvalidated fusion genes, Total= total discovered fusion-gene candidates, *P*-value = two-sided *p*-value of Fisher’s exact test of the difference in precision between FuSeq vs each of the other methods

We further evaluate whether the differences of the precision values of FuSeq to other methods are statistically significant. To do this, we collect the number of true positives (TP) and the total discovered fusion-gene candidates (Total) from Tables 1 and 2. Fisher’s exact test is then applied, comparing the precision between FuSeq vs each of the other methods. Two-sided *p*-values are reported in the tables. The results show that the tests for the precision difference between FuSeq and TopHat-Fusion are significant in the breast cancer datasets TP27 (9.1e-06) and TP99 (1.1e-08). For the Melanoma data, the test is significant vs SOAPfuse (*p*-value = 0.02). Moreover, in the Glioma datasets, the higher precision of FuSeq is significant vs two methods including JAFFA (5.5e-08) and SOAPfuse (0.02). There are no significant *p*-values vs any methods in sample SRR1659964; this likely due to the small sample problem. Finally, the higher precision of FuSeq is significant vs FusionMap (*p*-value = 0.04) and TopHat-Fusion (1.6e-22) in the full spike-in dataset.

### Evaluation by discovery rates

Since we do not have information on true negatives in the real datasets, we further evaluate the discovery rates of FuSeq through the ranks of validated fusion genes. In general, the validated fusion genes of each dataset should have low ranks in the final set. In the breast-cancer, melanoma and glioma datasets, most of the validated fusion genes are in the top 10 in the final set of a sample (Additional file 1: Table S1, S2 and S3). These fusion genes usually contains a high number of supporting reads (≥10).

Additional file 1: Figure S4 presents the ranks of 9 spike-in fusion genes over 20 samples detected by FuSeq. The samples in the x axis are ordered by the concentration levels from high to low and replication 2 to replication 1. In general, all spike-in fusions are on the top of ranks, indicating the stability of FuSeq. Half of samples in the right side, which have high levels of concentration, reveal all 9 spike-in fusion genes at the top 9 of ranks. This indicates that the spike-in fusion genes have the strong signals in these samples with high supporting reads (about more than 50 counts, see Additional file 1: Table S4). The trend of the ranks of spike-in fusion genes only slightly increases when the level of concentration decreases. The higher ranks of spike-in fusion genes in the low levels of concentration might be caused by higher signals of endogenous fusions in the baseline cell line. The more details of the ranks of each spike-in fusion genes crossing over 20 samples are reported in Additional file 1: Table S5. We also report candidates for endogenous fusion of the spike-in dataset in Additional file 1: Table S6. These endogenous fusion candidates are replicated at least a half of samples (10 times).

Furthermore, in order to compare to other fusion detection methods, we plot operating characteristic (OC) curves in Fig. 3. First, we extract the number of supporting reads of all fusion genes in the datasets. Then, we rank fusion genes and report the number of true positives discovered by FuSeq. For ranking, in a single sample, we sort fusion genes by their scores in decreasing order. To summarize the information from all samples of a dataset, we rank fusion genes by the maximum scores across the samples of the dataset. The results from FusionMap, TRUP, JAFFA, SOAPfuse and TopHat-Fusion are presented by crosses, circles, stars, diamonds and triangles in the plots.

For the breast-cancer and glioma datasets, we collect the list of ranked fusion genes from previous study [14] for JAFFA, SOAPfuse and TopHat-Fusion to build their OC curves. For melanoma and spike-in dataset, no ranking information of fusion gene available. The OC curve for all 20 samples of the spike-in dataset is not reported since we do not have information of the total number of fusion genes discovered by the these methods. As shown in the plots, the points of JAFFA, SOAPfuse and TopHat-Fusion generally follow the trends of the curves. However, FuSeq potentially discovers more true positives than JAFFA and TopHat-Fusion and less false positives than SOAPfuse. The OC curves of two methods TopHat-Fusion and SOAPfuse are worse than both JAFFA and FuSeq. In the glioma dataset, the OC curve of FuSeq is better than that of JAFFA. For the breast-cancer dataset, FuSeq are better and competitive with JAFFA at the beginning and the end of the OC curves. Moreover, in both TP27 and TP99 datasets, FuSeq discovers more true positives than JAFFA.

### Verification using de novo assembly

We illustrate the de novo assembly for one sample from each dataset. In each dataset, we select the sample with the highest number of validated fusion genes detected by FuSeq: SRR064439 (7.9M read-pairs) from the breast-cancer dataset, SRR018266 (14.9M read-pairs) from the melanoma dataset, and SRR934930 (28.7M read-pairs) from the glioma dataset. For the spike-in dataset, for consistency with previous works, we select sample SRR1659964 (93.9M read-pairs). The numbers of supporting read pairs from fusion-gene candidates used as input into Trinity software are 321, 165, 310 and 481 for the breast-cancer, melanoma, glioma and spike-in samples respectively. The results are summarized in Table 3.

**Table 3:** Verification of fusion genes by de novo assembly

		FuSeq	FuSeq + de novo assembly
Breast cancer	Total	22	4
	TP27	9	3
	TP99	16	4
Melanoma	Total	10	1
	TP	3	1
Glioma	Total	28	18
	TP	4	4
Spike-in	Total	25	12
	TP	9	9

TP= true positive fusion genes, Total= total discovered fusion-gene candidates

The numbers of fusion-gene candidates and the validated fusion genes discovered by methods are presented in rows Total and TP (true positive), respectively. The results in column ‘FuSeq + de novo assembly’ indicate the fusion-gene candidates supported by contigs from de novo assembly. For both breast-cancer and melanoma datasets, only a few fusion-gene candidates are verified by the assembly, but all of them are true positive. For the long read datasets (the glioma and spike-in datasets), all validated and spike-in fusion genes discovered by FuSeq are verified again by the de novo assembly. It also introduces the evidences of contigs from 14 and 3 extra fusion genes for the glioma dataset and the spike-in dataset, respectively. These examples show that de novo assembly increases the specificity of the fusion discovery, but a sufficient number of supporting reads is needed to achieve similar sensitivity as the mapping-based approach.

In general, de novo assembly is a useful step to add confidence in a fusion event if the fusion gene is associated with a contig. However, if the number of supporting reads are too low (low-abundant fusion genes), or there are no split read input, there might be not enough information to build a contig. Therefore, a true positive might not be supported by a contig. We can also use this result as an indication that in general a de novo assembly-based method is not a good detection method for low-abundant fusion genes.

### Computational time

**Fig. 4:**
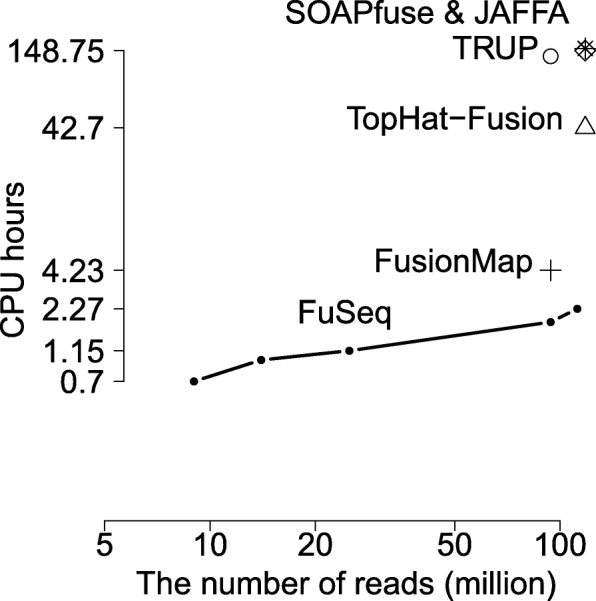
Comparison of fusion gene detection methods in computational time. Four methods JAFFA, SOAPfuse, TopHat-Fusion and FuSeq are compared according to the average computational time per sample over a range of sample sizes. Comparison results of TRUP and FusionMap and FuSeq for sample SRR1659964 from the spike-in dataset are located at ∼94M of the x-axis. Note that both axes are in log-scale

The computational cost of FuSeq comes from two steps including (i) quasi-mapping and (ii) statistical tests and filtering. The first step gets the benefit from the excellent efficient performances of the light-weight hash-table-based quasi-mapping process from Rapmap [26] for speed and memory usage. The time and memory of the second step is linear to the number of reads supporting the fusion genes candidates which is usually proportional to the sample’s library size. Figure 4 demonstrates that our method computational time is linear to the number of reads of samples. Similar to other methods, FuSeq keeps all the reads of fusion candidates that can be helpful for users. Therefore the storage requirements of FuSeq is also linear to the number of reads.

Due to the speed advantage of the quasi-mapping step [26] and the fusion equivalence class structure, FuSeq shows an excellent performance in computational time, see Fig. 4 and Additional file 1: Table S7. The time starts from the processing of the FASTQ file until the production of final candidates. For small datasets such as the breast-cancer and melanoma datasets, it takes 4-6 CPU hours to finish 6 samples (less than an hour per sample). It slightly increases to 1.15 h in average to process the long-read glioma dataset. It takes 45.47 CPU hours to complete all 20 samples of the big spike-in dataset (an average of 112M 100bp read-pairs per sample), an average of 2.27 CPU hours per sample.

We now compare this to other methods based on a recent report [9], where they compare 15 fusion detection methods for a single prostate cancer 171T sample (118M 100bp read-pairs). As reported, these methods require from 7.3 up to 240 h to analyse the sample. Among those, TopHat-Fusion, SOAPfuse and JAFFA need 42.7, 148.75 and 154 h respectively. Figure 4 presents the comparison between FuSeq and the three methods by the average computational time per sample over sample sizes. Thus, FuSeq provides a significant improvement (∼19, 66 and 68 times faster than TopHat-Fusion, SOAPfuse and JAFFA, respectively) in computational time.

We further compare performace times of FuSeq with FusionMap [15] known as the fastest methods in the comparison study, and TRUP [5] reported with similar total time compared to FusionMap and TopHat using their datasets. We select sample SRR1659964 containing 94 million reads and run three methods on this sample. The results show FuSeq requires 1.83 h, which is more than 2-fold faster than FusionMap (4.08 h), while TRUP requires a lot more time (136.04 h). These results are presented in Fig. 4 and details of computational time and memory usage are given in Additional file 1: Table S8.

## Discussion and conclusion

We have developed a novel method called FuSeq for fast and accurate discovery of fusion genes from RNA-seq data. The experiments of the method on four different real datasets with validated fusion genes reveal that FuSeq compares well against TopHat-Fusion, SOAPfuse and JAFFA in terms of sensitivity, specificity and F1 score. FuSeq also substantially improves on the computational time compared to the other fusion detection methods. Overall, FuSeq makes it easier to perform fusion gene discoveries from large RNA-seq datasets, e.g. involving large numbers of samples.

## Additional file

Additional file 1 Supplementary documents. (DOC 450 kb)

## Abbreviations

Bp: Base pair
FEQ: Set of fusion equivalence classes
Fge: Fusion gene
Ftx: Fusion transcript
FTX: set of fusion transcripts
FGE: set of fusion genes
MR: mapped read
OC: Operating characteristic
RNA-seq: RNA sequencing
SR: Split read
TP: true positive
