# Supplementary material for: A fast detection of fusion genes from paired-end RNA-seq data
Source: BMC Genomics. 2018 Nov 1;19:786. doi: 10.1186/s12864-018-5156-1 (PMC6211471; doi:10.1186/s12864-018-5156-1)
Supplement: Supplementary file 1 — Supplementary documents. (DOC 450 kb) [file 12864_2018_5156_MOESM1_ESM.doc]

Supplementary documents

A fast detection of fusion genes from paired-end RNA-seq data

By Trung Nghia Vu, et al

**Statistical tests and filtering of FuSeq**

This section presents more details of the statistical tests and filtering described in the main text. The values used for thresholds of the filtering steps are also included.

**Split read pipeline**

From quasi-mapping, we extract all split reads to build fusion gene candidate list. In general, each split read contains the information of mapping position in transcripts and reads, the length of mapped sequences and the mapping direction of the first and the last k-mers from the k-mer list as described in the figure S2. The *supportCount* of each fusion candidate is calculated by the number of split reads supporting the fusion gene. Then, various statistical tests and filters are applied to exclude false positives.

1. Chromosomes: the list of chromosomes can be input by user, by default we keep only fusion gene candidates of chromosomes 1-22, X and Y.

2. Expression of inverted fusion: if a fusion gene *fge*(*geneA, geneB*) is expressed, the inverted direction fusion gene *fge*(*geneB, geneA*) is not likely expressed. We allow less than 1% of expression of inverted fusion genes:

*supportCount*(*fge*(*geneB, geneA*)) *< supportCount*(*fge*(*geneA, geneB*))*/*100.

3. Distance between two constituent genes: if two genes are too close each other, their sequences have highly chance to be similar, thus the fusion gene candidate (*geneA, geneB*) is likely false positive. We allow the minimum distance is 100000 bases: distance(*geneA, geneB*) *>* 100000. This value can be set by users.

4. Overlapping sequence of the first and the last k-mer: Using the querypos and mapped len, we can compute the length of the overlapping mapped sequence between two genes in a split read. All fusion gene candidates at least one split read with the length is greater than 10 are put into a highly confident false positive list (FP list), and excluded from further analysis.

5. Fragment length test: From the mapping position of a split read and the left read in the read pair, the corresponding fragment length is calculated for statistical test. We exclude all split reads with fragment lengths out of 0.01% confident intervals of observed fragment length achieved from all other read pairs mapped to transcriptome.

6. Exon boundary and canonical splicing sites: We extract junction breaks of the split reads and checking the conditions of exon boundary and splicing sites from the databases. By default, all split reads not satisfying the exon boundary condition are excluded in FuSeq.

7. Paralogs: FuSeq removes all fusion gene candidates that two genes are paralogs. The information of paralogs is obtained from the database.

8. Misalignment: The corresponding sequences with the same read length of both transcripts in each fusion site are extracted and compared to the original read sequence. If 85% bases of the read sequence are fully mapped to either 3' transcript or 5' transcript, the split reads are definitely false positives.

9. The concordance of read direction and mapped direction (strand direction): remove fusion genes if there are at least 5\% split reads discordance (just applied for the data where the strand information is kept).

10. Junction distance: If two genes are from the same chromosome, a distance between two breaking points (*junctionDistance*) is calculated. If two junctions that are very close to each other, the fusion event is likely a false positive. We eliminate the fusion gene candidates with *junctionDistance ≤* 100000.

11. Positional distribution of split reads: From the mapped positions, the distributions of reads in the transcripts of each site are collected. For high support fusion genes (>=5), we keep fusion gene candidates with the standard deviation of the distribution must be no less than 1.

12. The consistency between mapped reads and split reads: We collect the corresponding mapped reads supporting the fusion gene candidates detected from the split reads using mapped read pipeline (see below). Then, we apply the same statistical tests and filter for estimated expression level of split reads and quasi-duplicated reads (items 9.3 and 10 in the mapped read pipeline below) to filter out false positives. In addition, we also check the consistency of breaking points from split reads and the estimated breaking points from mapped reads.

14. Ending exon of gene: The junction breaks at the 5’ site (3’ site) of the fusion must not be at the ending (starting) exon of the gene.

15. Site paralogs: Site paralogs occurs in two fusion genes sharing the same gene and breaking point in one fusion site while the genes of the other fusion site (partner site) are paralog or overlapping. If the two genes in the partner site are not paralog or overlapping, these fusion gene candidates are excluded.

16. Supporting read count: by default, we keep only fusion genes with supporting reads *≥* 1.

**Mapped read pipeline**

After generating the fusion candidates from fusion equivalence classes (Figure S2), we apply different statistical tests and filtering to eliminate false positives. Since the mapped reads might not contain junction break which is the strong evidence for fusion event, statistical tests and filtering in mapped read pipeline are generally more stringent.

1. Chromosomes: similar to the split read pipeline, we keep only fusion gene candidates of chromosomes 1-22, X and Y.
2. Fusion equivalence class sharing: We do not expect many supporting read pairs shared between fusion genes. In practice, we allow maximum 5% sharing counts:

(1 *− correctedCount*(*fgek*) */ supportCount*(*fgek*)) *≤* 0*.*05.

3. Expression of inverted fusion: the same method in the split read pipeline is applied for the mapped reads.

4. Distance between two constituent genes: the same method in the split read pipeline is applied for the mapped reads.
5. Multiple-fusion genes: we set *maxFusionNum* as the maximum number of fusion events occurring in a single gene. We also consider fusion genes with more than *sgtCount* supporting fragments as a strong signal. The default settings of these values in our method are 2 for *maxFusionNum* and 10 for *sgtCount*. Then, several filters are used:

1. Eliminate fusion genes that there are more than *maxFusionNum* fusions with supporting fragments *> sgtCount*.
2. Eliminate fusion gene (*geneA, geneB*) where
   *supportCount*(*geneA, geneB*) *≤ maxFusionFc ∗ supportCount*(*geneA, geneX* )
   or
   *supportCount*(*geneA, geneB*) *≤ maxFusionFc ∗ supportCount*(*geneY , geneB*),
   where (*geneA, geneX* ) is not in top *maxFusionNum* fusion genes associated with *geneA* at 5 prime, similarly for
   (*geneY , geneB*) with *geneB* at 3 prime. The value of the fold change *maxFusionFc* is 2 by default in our pipeline.
3. Keep top *maxFusionNum* fusion genes for a single constituent gene.

6. Supporting read counts: by default, we keep only fusion genes with supporting fragments *≥* 2.

7. Protein-coding genes: we keep only fusion genes that their constituent genes are protein-coding genes.

8. Sequence similarity of constituent genes
In fact, different genes with highly similar sequences are easily listed in fusion gene candidates but they are likely false positive. Because there might not evident of junction break in mapped reads, we cannot make sure these candidates are true positive or not. Therefore, in mapped read pipeline, we exclude all candidates with evidences of sequence similarity. The similarity is frequently observed between a gene and its paralogs or it known read-through (conjoined) genes. We also apply the filter for the gene pair with similar sequences detected from equivalence class as described in the main text.
9. Distribution of supporting reads of fusion gene
For each read pair (*r*1*, r*2), we collect all start positions of *r*1 and *r*2 mapped to the annotation reference. Thus, for each fusion gene (*geneA, geneB*), we are able to compute the distributions of the start positions (*startPos*) of the supporting reads to *geneA* and *geneB*. For simplicity, we call them positional distribution. From the distributions of two sites, we estimate the fragment lengths (contribution from each sites and combination) that the read pairs are originally generated. Since we have observed fragment length distribution from all other read pairs mapped to transcriptome, we are able to refine the supporting reads of gene fusions by detecting and excluding outliers using the estimated fragment length distribution. In particular, extract the variances to the mean of estimated fragments of each site and keep only ones with the value higher than 0.01% confidence from the variances of observed fragments. From the positional distribution of k-mer mappings, we can get the chromosome positions of the reads and exons containing the positions. If exon boundary and canonical sites are required, the information of exon ends can be used to estimate the breakpoints. However, the information from split reads is better for breakpoints.

9. 1. Statistical tests for the estimated fragment length:

- Median of estimated fragment length of fusion gene must be greater than the length of read and in the range observed fragment length distribution (99.99 % observed fragment lengths).

- We compute an empirical p-value for mean of estimated fragment length of each site using observed fragment length. We filter out fusion gene candidates with the p-value less than 0.10.

9.2. Removal of duplicated read pairs: Thus, information of read distribution allows determining the number of duplicated fragments (*dupCount*) supporting the fusion gene. After removing duplicated read pairs, the non-duplicated supporting count is calculated as *nondupCount* = *supportCount − dupCount*. In our pipeline, we keep fusion genes with *nondupCount ≥* 2. 9.2.

9.3 Removal of quasi-duplicated reads: If the variance of positional distribution is not greater than 1, we consider the reads are quasi-duplicated and exclude the fusion gene candidate if it has more than 3 supporting reads.

9.4. Junction distance: If two genes are from the same chromosome, from the distribution of start position, the distance between two distributions of *gene*5 and *gene*3 is calculated (*junctionDistance*). Two junctions that are very close to each other likely indicate a false positive fusion event. We eliminate the fusion gene candidates with *junctionDistance ≤* 100000.

10. Expression level of split reads: From the positional distribution of reads, we extract the minimum set of exons explaining the all the reads to estimate the length of fusion transcripts flen5 and flen3 for three prime and five prime sites respectively. We can estimate the expression level of split reads from 5’ site (similarly to 3’ site) by estSR5=supportCount/flen5*(r-2*k) where r is read length and k is k-mer length. Since in FuSeq, the split read range in very small region (r-2*k), we assume the uniform distribution for the reads. If the corresponding split reads of the fusion candidate are expressed (>0.001 mapped read supporting counts), we expect the number of observed split reads is higher than the estimated split reads, so exclude all the conflicted cases.

11. False positive list from split read pipeline: We also exclude all fusion gene candidates appearing in the high confident false positive list (FP list) in the split read pipeline.

**Figures and Tables**

**
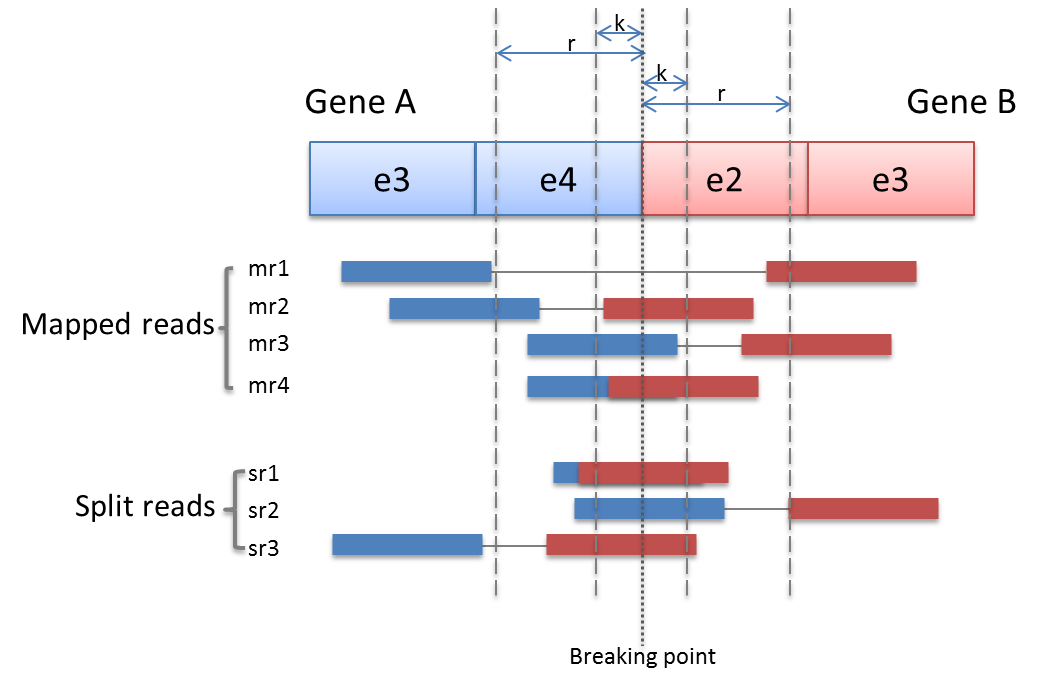
**

**Figure S1.** Mapped reads and split reads in FuSeq. In mapped reads, at least (*r*-*k*-1) bases of a read must be mapped to a transcript (gene). In split reads, at least *k* bases of a read must be mapped to each transcript of the genes, and the other read of the read pair is mapped to either transcripts. In the figure, *r* and *k* is the length of read and k-mer respectively.

**
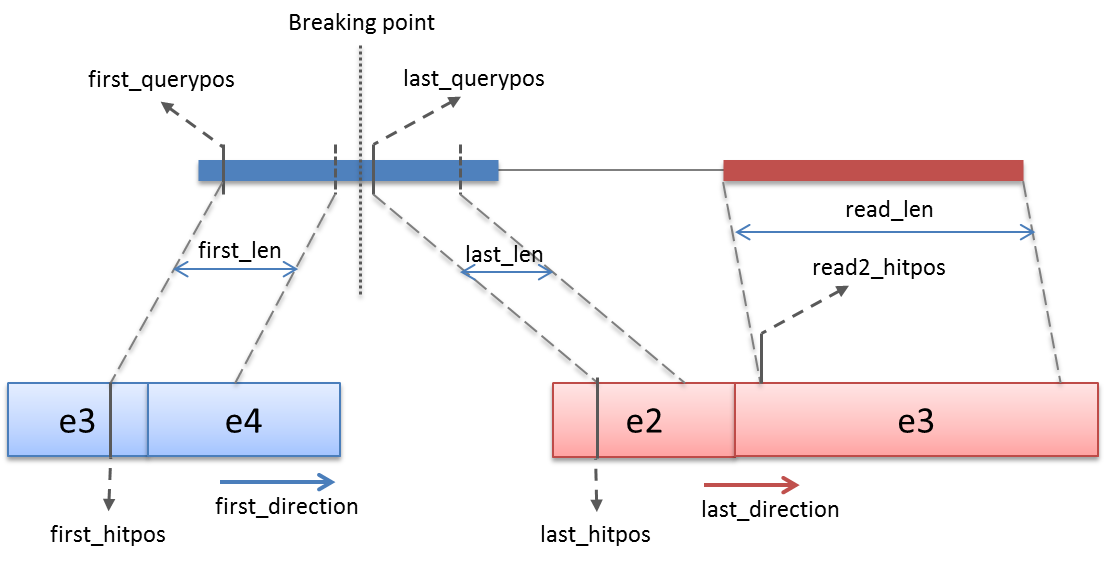
**

**Figure S2.** Information extraction for split reads. In this figure, “first” and “last” represent the first and the last k-mer mapping from a read. In each k-mer, query position (the relative position of the k-mer in the read), hitpos (the mapped position of the k-mer sequence in the transcript), mapped sequence length and mapped direction are reported. Moreover, the other read of the read pair must be mapped to the same transcripts of the either fusion sites; and its mapping information including hitpos and mapped direction is also reported.

**
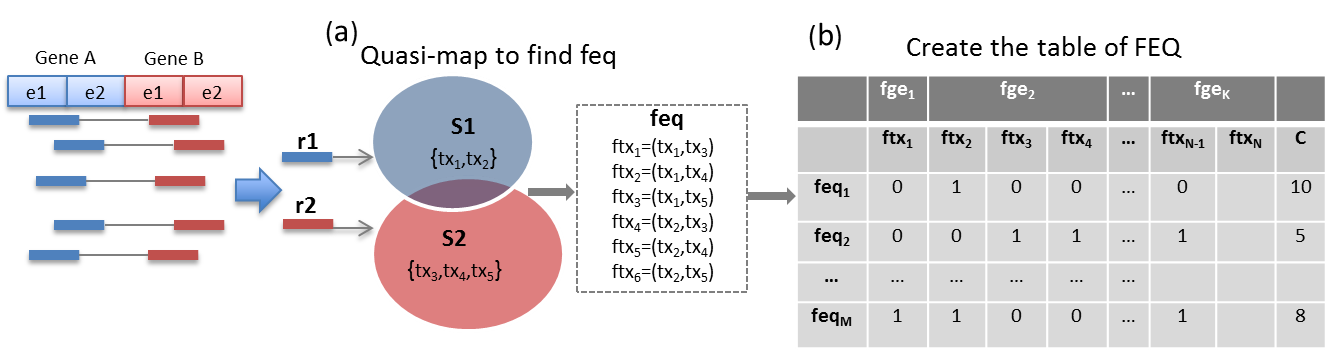
**

**Figure S3.** Generation of fusion equivalence classes: (a) quasi-mapping of read pairs to determine fusion equivalence classes (feq); (b) build the FEQ table, the collection of feq's, and extract initial fusion gene candidates.

**
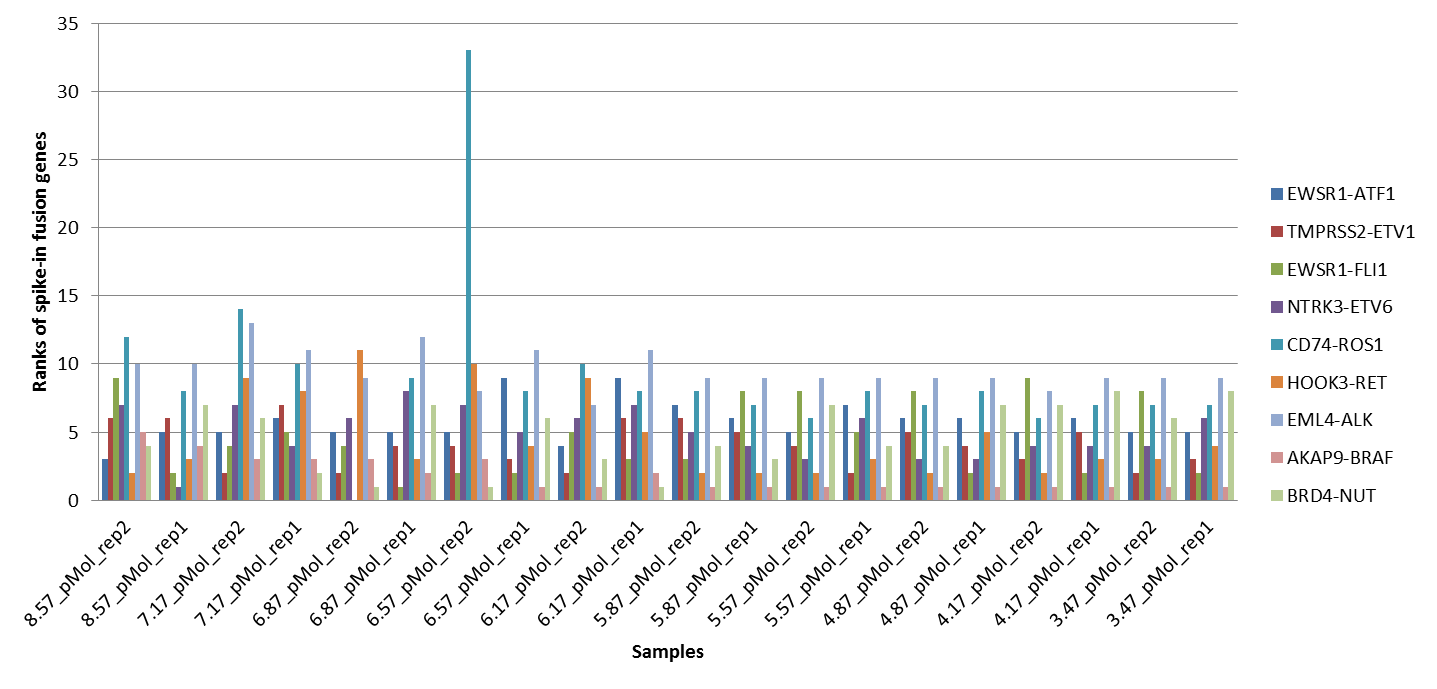
**

**Figure S4.** Ranks of spike-in fusion genes discovered by FuSeq. The x-axis gives the names of the samples, for example 8.57_pMol_rep2, which includes the level of concentration in log10scale (-8.57 log10(pMol)) and replication index (replication 2).

**Table S1.** Rank and number of supporting reads of the breast cancer using 27 validated fusion genes. The reports for BT-474 and SKBR3 cell lines include a single sample and another one in parentheses. In particular, the order format of the samples for cell line BT-474 is SRR064438(SRR064439) and cell line SKBR3 is SRR064440(SRR064441).

| | **Fusion gene** | **Supporting reads** | **Rank** | **Total** | **SampleID** | | --- | --- | --- | --- | --- | | ACACA-STAC2 | 46(66) | 1(1) | 20(22) | BT-474 | | RPS6KB1-SNF8 | 26(35) | 2(4) | _ | BT-474 | | VAPB-IKZF3 | 24(40) | 3(2) | _ | BT-474 | | ZMYND8-CEP250 | 17(32) | 7(5) | _ | BT-474 | | RAB22A-MYO9B | 12(9) | 9(9) | _ | BT-474 | | SKA2-MYO19 | 7(NA) | 17(NA) | _ | BT-474 | | DIDO1-KIAA0406 | 5(7) | 15(11) | _ | BT-474 | | STARD3-DOK5 | 5(5) | 14(12) | _ | BT-474 | | LAMP1-MCF2L | NA(5) | NA(13) | _ | BT-474 | | GLB1-CMTM7 | 4(5) | 19(15) | _ | BT-474 | | CPNE1-PI3 | NA(NA) | NA(NA) | _ | BT-474 | | TATDN1-GSDMB | 267(404) | 1(1) | 9(12) | SKBR3 | | CSE1L-ENSG00000236127 | NA(NA) | NA(NA) | _ | SKBR3 | | RARA-PKIA | 10(13) | 5(3) | _ | SKBR3 | | ANKHD1-PCDH1 | 8(10) | 7(6) | _ | SKBR3 | | CCDC85C-SETD3 | NA(NA) | NA(NA) | _ | SKBR3 | | SUMF1-LRRFIP2 | 12(10) | 4(4) | _ | SKBR3 | | WDR67-ZNF704 | NA(3) | NA(10) | _ | SKBR3 | | CYTH1-EIF3H | 18(53) | 3(2) | _ | SKBR3 | | DHX35-ITCH | NA(4) | NA(8) | _ | SKBR3 | | NFS1-PREX1 | NA(NA) | NA(NA) | _ | SKBR3 | | BSG-NFIX | 37 | 1 | 9 | KPL-4 | | PPP1R12A-SEPT10 | 6 | 4 | _ | KPL-4 | | NOTCH1-NUP214 | 11 | 2 | _ | KPL-4 | | BCAS4-BCAS3 | 202 | 1 | 8 | MCF-7 | | ARFGEF2-SULF2 | 32 | 2 | _ | MCF-7 | | RPS6KB1-TMEM49 | NA | NA | _ | MCF-7 | |  |  |  |
| --- | --- | --- | --- | --- | --- | --- | --- | --- | --- | --- | --- | --- | --- | --- | --- | --- | --- | --- | --- | --- | --- | --- | --- | --- | --- | --- | --- | --- | --- | --- | --- | --- | --- | --- | --- | --- | --- | --- | --- | --- | --- | --- | --- | --- | --- | --- | --- | --- | --- | --- | --- | --- | --- | --- | --- | --- | --- | --- | --- | --- | --- | --- | --- | --- | --- | --- | --- | --- | --- | --- | --- | --- | --- | --- | --- | --- | --- | --- | --- | --- | --- | --- | --- | --- | --- | --- | --- | --- | --- | --- | --- | --- | --- | --- | --- | --- | --- | --- | --- | --- | --- | --- | --- | --- | --- | --- | --- | --- | --- | --- | --- | --- | --- | --- | --- | --- | --- | --- | --- | --- | --- | --- | --- | --- | --- | --- | --- | --- | --- | --- | --- | --- | --- | --- | --- | --- | --- | --- | --- | --- | --- | --- | --- |

**Table S2.** Rank and number of supporting reads of the melanoma dataset

| | **Fusion gene** | **Supporting reads** | **Rank** | **Total** | **Sample** | | --- | --- | --- | --- | --- | | KCTD2-ARHGEF12 | 6 | 2 | 3 | M000216 | | ANKHD1-C5orf32 | NA | NA | 4 | M990802 | | RB1-ITM2B | NA | NA | _ | M990802 | | GCN1L1-PLA2G1B | 5 | 1 | 1 | M980409 | | SCAMP2-WDR72 | 5 | 1 | 2 | M010403 | | CCT3-C1orf61 | NA | NA | 10 | 501-MEL | | GNA12-SHANK2 | 36 | 3 | _ | 501-MEL | | PARP1-MIXL1 | 6 | 7 | _ | 501-MEL | | SLC12A7-C11orf67 | 68 | 1 | _ | 501-MEL | | RECK-ALX3 | 27 | 1 | 4 | M000921 | | TMEM8B-TLN1 | NA | NA | _ | M000921 | |  |  |  |  |
| --- | --- | --- | --- | --- | --- | --- | --- | --- | --- | --- | --- | --- | --- | --- | --- | --- | --- | --- | --- | --- | --- | --- | --- | --- | --- | --- | --- | --- | --- | --- | --- | --- | --- | --- | --- | --- | --- | --- | --- | --- | --- | --- | --- | --- | --- | --- | --- | --- | --- | --- | --- | --- | --- | --- | --- | --- | --- | --- | --- | --- | --- | --- | --- | --- |

**Table S3.** Rank and number of supporting reads of the glioma dataset

| **Fusion gene** | **Supporting reads** | **Rank** | **Total** | **Sample** |
| --- | --- | --- | --- | --- |
| CBL-FBXO2 | 5 | 10 | 24 | SRR934744 |
| MGAT5-KIAA0825 | 5 | 5 | _ | SRR934744 |
| TTLL11-FIBCD1 | 5 | 8 | _ | SRR934744 |
| AHCYL2-TMEM178B | 36 | 1 | 16 | SRR934746 |
| CLASRP-SYMPK | 19 | 2 | _ | SRR934746 |
| FRMD4A-PFKP | 9 | 6 | 19 | SRR934774 |
| MRPS28-ASPH | 14 | 8 | _ | SRR934774 |
| KMT2C-CHGB | 74 | 2 | 15 | SRR934868 |
| VHL-BRK1 | NA | NA | _ | SRR934868 |
| AP2A2-SBF2 | 1176 | 2 | 14 | SRR934871 |
| CD81-SPAG6 | 344 | 3 | _ | SRR934871 |
| RASSF7-SCUBE2 | 22 | 7 | _ | SRR934871 |
| CCM2-OGDH | 10 | 4 | 12 | SRR934875 |
| TPM3-ADAR | 31 | 2 | _ | SRR934875 |
| GPR162-CCDC39 | 14 | 3 | 22 | SRR934887 |
| SLC6A8-GABRA3 | 83 | 1 | _ | SRR934887 |
| B4GALNT3-DENND5B | 48 | 3 | 28 | SRR934902 |
| MED13L-GRIP1 | 134 | 2 | _ | SRR934902 |
| BRPF3-CLPSL1 | 101 | 2 | 16 | SRR934915 |
| FAM155A-COL4A1 | 219 | 1 | _ | SRR934915 |
| PVRL2-SNTG1 | 20 | 1 | 12 | SRR934918 |
| ST7-CTTNBP2 | 4 | 8 | _ | SRR934918 |
| C15orf57-CBX3 | 4 | 6 | 7 | SRR934929 |
| CDK17-KCNC2 | 13 | 2 | _ | SRR934929 |
| COG3-GPC6 | 13 | 5 | 28 | SRR934930 |
| LHFP-SERP2 | 8 | 8 | _ | SRR934930 |
| TCF7L1-KIF1B | NA | NA | _ | SRR934930 |
| URI1-SLC6A20 | 15 | 4 | _ | SRR934930 |
| ZMIZ1-MAT1A | 66 | 1 | _ | SRR934930 |
| TPT1-AADAT | 24 | 1 | 13 | SRR934947 |
| ZNF197-CHMP2B | 13 | 2 | _ | SRR934947 |

**Table S4.** Overall supporting counts of spike-in fusion genes discovered by FuSeq. The name of sample in each row, for example 3.47_pMol_rep1, includes the level of concentration in log10 scale (-3.47 log10(pMol)) and replication index (replication 1).

|  | **EWSR1-ATF1** | **TMPRSS2-ETV1** | **EWSR1-FLI1** | **NTRK3-ETV6** | **CD74-ROS1** | **HOOK3-RET** | **EML4-ALK** | **AKAP9-BRAF** | **BRD4-NUT** |
| --- | --- | --- | --- | --- | --- | --- | --- | --- | --- |
| **8.57_pMol_rep2** | 12 | 10 | 8 | 10 | 4 | 13 | 7 | 11 | 11 |
| **8.57_pMol_rep1** | 34 | 30 | 44 | 51 | 14 | 38 | 12 | 37 | 29 |
| **7.17_pMol_rep2** | 15 | 25 | 18 | 13 | 5 | 8 | 6 | 19 | 13 |
| **7.17_pMol_rep1** | 14 | 14 | 14 | 21 | 9 | 13 | 6 | 16 | 19 |
| **6.87_pMol_rep2** | 13 | 39 | 22 | 13 | *NA* | 8 | 9 | 39 | 42 |
| **6.87_pMol_rep1** | 15 | 16 | 25 | 17 | 12 | 21 | 5 | 22 | 14 |
| **6.57_pMol_rep2** | 19 | 27 | 29 | 12 | 3 | 7 | 10 | 30 | 47 |
| **6.57_pMol_rep1** | 15 | 32 | 35 | 37 | 18 | 30 | 8 | 37 | 23 |
| **6.17_pMol_rep2** | 60 | 147 | 44 | 37 | 14 | 23 | 37 | 187 | 216 |
| **6.17_pMol_rep1** | 24 | 40 | 45 | 51 | 25 | 42 | 15 | 62 | 67 |
| **5.87_pMol_rep2** | 103 | 127 | 148 | 157 | 64 | 159 | 59 | 183 | 133 |
| **5.87_pMol_rep1** | 111 | 125 | 142 | 171 | 69 | 152 | 38 | 160 | 147 |
| **5.57_pMol_rep2** | 312 | 332 | 416 | 471 | 209 | 413 | 152 | 532 | 361 |
| **5.57_pMol_rep1** | 209 | 269 | 258 | 313 | 116 | 262 | 95 | 295 | 265 |
| **4.87_pMol_rep2** | 1106 | 1108 | 1436 | 1738 | 636 | 1277 | 454 | 1600 | 1231 |
| **4.87_pMol_rep1** | 1069 | 1192 | 1324 | 1590 | 556 | 1158 | 441 | 1415 | 1287 |
| **4.17_pMol_rep2** | 8699 | 9028 | 10551 | 11464 | 5532 | 10213 | 4144 | 13066 | 10254 |
| **4.17_pMol_rep1** | 8372 | 8526 | 10275 | 11445 | 5099 | 9613 | 3498 | 13198 | 9010 |
| **3.47_pMol_rep2** | 49252 | 60369 | 61494 | 67146 | 27053 | 55598 | 21892 | 71424 | 60385 |
| **3.47_pMol_rep1** | 43805 | 47442 | 53203 | 55576 | 23958 | 44485 | 17584 | 56743 | 46738 |

**Table S5.** Overall ranks of spike-in fusion genes discovered by FuSeq. The annotations of row names are the same as Table S4.

| |  | **EWSR1-ATF1** | **TMPRSS2-ETV1** | **EWSR1-FLI1** | **NTRK3-ETV6** | **CD74-ROS1** | **HOOK3-RET** | **EML4-ALK** | **AKAP9-BRAF** | **BRD4-NUT** | **Total** | | --- | --- | --- | --- | --- | --- | --- | --- | --- | --- | --- | | **8.57_pMol_rep2** | 3 | 6 | 9 | 7 | 12 | 2 | 10 | 5 | 4 | 20 | | **8.57_pMol_rep1** | 5 | 6 | 2 | 1 | 8 | 3 | 10 | 4 | 7 | 30 | | **7.17_pMol_rep2** | 5 | 2 | 4 | 7 | 14 | 9 | 13 | 3 | 6 | 44 | | **7.17_pMol_rep1** | 6 | 7 | 5 | 4 | 10 | 8 | 11 | 3 | 2 | 25 | | **6.87_pMol_rep2** | 5 | 2 | 4 | 6 | *NA* | 11 | 9 | 3 | 1 | 42 | | **6.87_pMol_rep1** | 5 | 4 | 1 | 8 | 9 | 3 | 12 | 2 | 7 | 23 | | **6.57_pMol_rep2** | 5 | 4 | 2 | 7 | 33 | 10 | 8 | 3 | 1 | 33 | | **6.57_pMol_rep1** | 9 | 3 | 2 | 5 | 8 | 4 | 11 | 1 | 6 | 26 | | **6.17_pMol_rep2** | 4 | 2 | 5 | 6 | 10 | 9 | 7 | 1 | 3 | 51 | | **6.17_pMol_rep1** | 9 | 6 | 3 | 7 | 8 | 5 | 11 | 2 | 1 | 27 | | **5.87_pMol_rep2** | 7 | 6 | 3 | 5 | 8 | 2 | 9 | 1 | 4 | 21 | | **5.87_pMol_rep1** | 6 | 5 | 8 | 4 | 7 | 2 | 9 | 1 | 3 | 45 | | **5.57_pMol_rep2** | 5 | 4 | 8 | 3 | 6 | 2 | 9 | 1 | 7 | 28 | | **5.57_pMol_rep1** | 7 | 2 | 5 | 6 | 8 | 3 | 9 | 1 | 4 | 21 | | **4.87_pMol_rep2** | 6 | 5 | 8 | 3 | 7 | 2 | 9 | 1 | 4 | 30 | | **4.87_pMol_rep1** | 6 | 4 | 2 | 3 | 8 | 5 | 9 | 1 | 7 | 27 | | **4.17_pMol_rep2** | 5 | 3 | 9 | 4 | 6 | 2 | 8 | 1 | 7 | 20 | | **4.17_pMol_rep1** | 6 | 5 | 2 | 4 | 7 | 3 | 9 | 1 | 8 | 21 | | **3.47_pMol_rep2** | 5 | 2 | 8 | 4 | 7 | 3 | 9 | 1 | 6 | 35 | | **3.47_pMol_rep1** | 5 | 3 | 2 | 6 | 7 | 4 | 9 | 1 | 8 | 31 | |  |  |  |  |  |  |  |  |  |
| --- | --- | --- | --- | --- | --- | --- | --- | --- | --- | --- | --- | --- | --- | --- | --- | --- | --- | --- | --- | --- | --- | --- | --- | --- | --- | --- | --- | --- | --- | --- | --- | --- | --- | --- | --- | --- | --- | --- | --- | --- | --- | --- | --- | --- | --- | --- | --- | --- | --- | --- | --- | --- | --- | --- | --- | --- | --- | --- | --- | --- | --- | --- | --- | --- | --- | --- | --- | --- | --- | --- | --- | --- | --- | --- | --- | --- | --- | --- | --- | --- | --- | --- | --- | --- | --- | --- | --- | --- | --- | --- | --- | --- | --- | --- | --- | --- | --- | --- | --- | --- | --- | --- | --- | --- | --- | --- | --- | --- | --- | --- | --- | --- | --- | --- | --- | --- | --- | --- | --- | --- | --- | --- | --- | --- | --- | --- | --- | --- | --- | --- | --- | --- | --- | --- | --- | --- | --- | --- | --- | --- | --- | --- | --- | --- | --- | --- | --- | --- | --- | --- | --- | --- | --- | --- | --- | --- | --- | --- | --- | --- | --- | --- | --- | --- | --- | --- | --- | --- | --- | --- | --- | --- | --- | --- | --- | --- | --- | --- | --- | --- | --- | --- | --- | --- | --- | --- | --- | --- | --- | --- | --- | --- | --- | --- | --- | --- | --- | --- | --- | --- | --- | --- | --- | --- | --- | --- | --- | --- | --- | --- | --- | --- | --- | --- | --- | --- | --- | --- | --- | --- | --- | --- | --- | --- | --- | --- | --- | --- | --- | --- | --- | --- | --- | --- | --- | --- | --- | --- | --- | --- |

**Table S6.** Endogenous fusion genes from the spike-in dataset discovered by FuSeq

| **No.** | **GeneA** | **GeneB** | **Median expression** | **Repeat times** |
| --- | --- | --- | --- | --- |
| 1 | AMDHD1 | DNAJC25 | 18 | 18 |
| 2 | C15orf57 | CBX3 | 5 | 15 |
| 3 | SCRN3 | C3orf67 | 5 | 13 |
| 4 | LYST | SPATA21 | 3 | 11 |

**Tabe S7.** Average computational time (in CPU hours) per sample for FuSeq fusion discovery in four different datasets; the time starts from the processing of the FASTA file until the production of final candidates.

|  | **Ave number of read pairs (million)** | **Read length** | **Time**  **(CPU hours)** |
| --- | --- | --- | --- |
| **Breast cancer** | 9 | 50bp | 0.70 |
| **Melanoma** | 14 | 50bp | 0.99 |
| **Glioma** | 25 | 101bp | 1.15 |
| **Spike-in** | 112 | 100bp | 2.27 |

**Table S8.** Computational time (in CPU hours) of sample SRR1659964 with 94M reads from the spike-in dataset for FusionMap, TRUP and FuSeq.

|  | **Time**  **(CPU hours)** | **Memory of usage (Gb)** |
| --- | --- | --- |
| **FuSeq** | 1.83 | 7.0 |
| **FusionMap** | 4.08 | 0.9 |
| **TRUP** | 136.04 | 3.8 |
